# Effects and predictors of shoulder muscle massage for patients with posterior shoulder tightness

**DOI:** 10.1186/1471-2474-13-46

**Published:** 2012-03-27

**Authors:** Jing-lan Yang, Shiau-yee Chen, Ching-Lin Hsieh, Jiu-jenq Lin

**Affiliations:** 1 Department of Physical Medicine & Rehabilitation, National Taiwan University Hospital, Taipei, Taiwan; 2 Department of Physical Medicine and Rehabilitation, Taipei Medical University-Municipal Wan Fang Hospital, Taipei, Taiwan; 3 School of Occupational Therapy, College of Medicine, National Taiwan University, Taipei, Taiwan; 4 School and Graduate Institute of Physical Therapy, College of Medicine, National Taiwan University, Taipei, Taiwan; 5 Adjunct Physical Therapist, Physical Therapy Center, National Taiwan University Hospital, Floor 3, No. 17, Xuzhou Rd, Zhongzheng District, Taipei City 100, Taiwan

**Keywords:** Massage, Stiff shoulder, Range of motion

## Abstract

**Background:**

Clinical approaches like mobilization, stretching, and/or massage may decrease shoulder tightness and improve symptoms in subjects with stiff shoulders. We investigated the effect and predictors of effectiveness of massage in the treatment of patients with posterior shoulder tightness.

**Methods:**

A randomized controlled trial was conducted in a hospital-based outpatient practice (orthopedic and rehabilitation). Forty-three women and 17 men (mean age = 54 years, range 43-73 years) with posterior shoulder tightness participated and were randomized into massage and control groups (n = 30 per group). A physical therapist provided the massage on the posterior deltoid, infraspinatus, and teres minor of the involved shoulder for 18 minutes [about 6 minutes for each muscle] two times a week for 4 weeks. For the control group, one therapist applied light hand touch on the muscles 10 minutes two times a week for 4 weeks. Glenohumeral internal rotation ROM, functional status, and muscle tightness were the main outcomes. Additionally, the potential factors on the effectiveness of massage were analyzed by multivariate logistic regression. For this analysis, patients with functional score improvement at least 20% after massage were considered responsive, and the others were considered nonresponsive.

**Results:**

Fifty-two patients completed the study (29 for the massage and 23 for the control). The overall mean internal rotation ROM increased significantly in the massage group compared to the control (54.9° v.s. 34.9°; *P *≤ 0.001). There were 21 patients in the responsive group and 8 in the nonresponsive group. Among the factors, duration of symptoms, functional score, and posterior deltoid tightness were significant predictors of effectiveness of massage.

**Conclusions:**

Massage was an effective treatment for patients with posterior shoulder tightness, but was less effective in patients with longer duration of symptoms, higher functional limitation, and less posterior deltoid tightness.

**Trial registration:**

This clinical trial is registered at Trial Registration "Trial registration: Clinicaltrials.gov NCT01022827".

## Background

Various theories exist regarding the mechanisms of stiff shoulder (SS). Potential etiological factors are adhesive capsulitis [1], decreased capsular volume [2,3], capsular contractions [4], rotator interval thickening and fibrosis[5], and subscapularis tendon thickening [5]. Cyriax [6] proposed that stiffness in a shoulder joint capsule would restrict motion in a predictable pattern, a capsular pattern in which external rotation is more limited than abduction, which in turn is more limited than internal rotation. Others authors have indicated that posterior shoulder stiffness is significantly correlated with humeral internal rotation ROM loss [7-9]. Specifically, several researchers [10-13] hypothesized that the stiffness of specific muscles (rotator cuff) may contribute to posterior shoulder stiffness. These potential mechanisms provide rationales for treatment protocols options.

Mobilization, stretching, and/or massage are advocated for patients with posterior shoulder tightness and limited glenohumeral internal rotation ROM [7,9,11,14]. Tightness in the posterior shoulder has been associated with a loss of glenohumeral internal rotation range of motion (ROM) [7,9,11]. It has been found that in cadaver models, tightness in the posterior shoulder has limited glenohumeral internal rotation ROM [7]. In subjects with subacromial impingement syndrome and frozen shoulder syndrome, decreased glenohumeral internal rotation ROM are related to posterior shoulder tightness [9,14,15]. Additionally, tightening of the posterior portion of the shoulder is associated with increased anterior and superior humeral head translations on the glenoid, which has been theorized to contribute to shoulder impingement syndrome [7,16]. Presumably, clinical approaches like mobilization, stretching, and/or massage may decrease shoulder tightness and improve symptoms in subjects with SS.

Although soft tissue massage of the posterior shoulder tissues is often included in rehabilitation of individuals with posterior shoulder tightness, glenohumeral internal rotation ROM deficit, and/or impingement syndrome, evidence to support treatment protocols is limited. Based on a case report, Poser and Casonato [17] suggest that massaging the infraspinatus and teres minor muscles can result in 20 degrees of improvement of internal rotation. However, they did not provide the rationale for different treatment durations for each muscle (7 minutes for the infraspinatus and 3 minutes for the teres minor). It is possible that various muscles may respond differently according to the massage technique. Additionally, improvement of internal rotation cannot reflect the tightness property of each specific muscle after massage. Since the effects of massage on muscle and connective tissue were based on ROM measurement in the majority of studies [18-20], the effect of massage on specific muscle tightness is not clear. This is important for clinicians to precisely target the involved anatomical structure (muscle or capsule) that is the source of the joint restriction.

The purpose of this study was to investigate the effect and predictors of effectiveness of massage in the treatment of patients with posterior shoulder tightness. We hypothesized that massage may effectively improve glenohumeral internal rotation in subjects with posterior shoulder tightness, and identify the predictors of effective massage by investigating the characteristics of the responsive subjects. This may help clinicians to decide whether massage is a worthy treatment for a patient with loss of internal rotation and posterior shoulder tightness.

## Methods

This was a randomized controlled study approved by the institutional review board of National Taiwan University Hospital (ClinicalTrials.gov ID: NCT01022827; Protocol ID: 200905041R). Patients evaluated as having glenohumeral internal rotation limitation in our outpatient clinic were eligible for participation in the study. The inclusion criteria were: (1) limitation of internal rotation ROM compared to the sound side at least 10%; (2) tightness in the posterior shoulder region. Posterior shoulder tightness was defined as more tightness measurement values at least 10% compared to the sound side. Measurement of posterior shoulder tightness was based on horizontal flexion ROM (cross-chest adduction) measurement [15]. Because we measured the transverse tightness of the muscles by myotonometer, skin/subcutaneous tissue thickness may affect the validity of the measurement. Thus, subjects with body mass index (BMI) (less than 19 or more than 24) were expected to have confounding factor of skin/subcutaneous tissue thickness on the muscle tightness measurement and were excluded from the study. The BMI was calculated by dividing his or her body weight in kilograms by the square of the body height in meters. The other exclusion criteria were: (1) surgery on the particular shoulder, (2) rheumatoid arthritis, (3) stroke with residual shoulder involvement, or (4) fracture of the shoulder complex.

Based on the judgment of what constitutes clinically meaningful differences and variability estimates from previous studies [7-9,17], a sample size of 25 subjects per group provided 80% power to detect differences of 15 degrees internal rotation ROM between the pre- and post-intervention as well as between the 2 groups of interest at an alpha level of .05 with a two-tailed test. They received a written and verbal explanation of the purposes and procedures of the study. If they agreed to participate, they signed informed consent forms approved by the Human Subjects Committee of NTUH.

**Figure 1:**
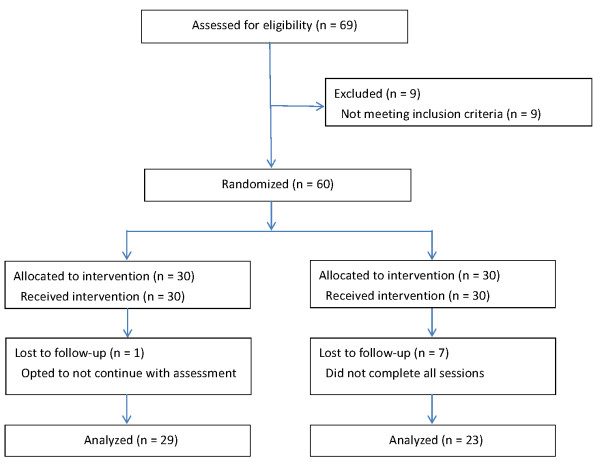
**CONSORT diagram of enrollment and flow of activities through the clinical trial**.

A total of 69 patients were recruited, of whom 9 were excluded by the criteria. Sixty patients were randomized by computer generated permuted block randomization of 15 by sequentially numbered, sealed, opaque envelopes to massage and control groups: 43 women and 17 men, with an average age of 54 years (range 43-73 years) (Table 1). The permutation lists were MMCC, MCMC, MCCM, CCMM, CMCM, CMMC (M: massage; C: control). Patients signed an informed consent form before participating in the study. Figure 1 presents a CONSORT diagram that summarizes the flow of activities and participants through the clinical trial.

**Table 1:** Subject demographics

variable	Massage (n = 29)	Control (n = 23)
Gender (males:females)	8:21	6:17

Age (years)	54.8 ± 8.5	54.6 ± 7.9

Height (cm)	165.4 ± 5.8	163.8 ± 9.8

Weight (Kg)	65.3 ± 5.9	66.3 ± 5.7

Duration of symptoms (months)	14.8 ± 8.4	15.7 ± 7.8

Pre^a^- Glenohumeral Internal rotation (°)	31.9 ± 11.2	28.7 ± 5.8

Post^a^- Glenohumeral Internal rotation (°)	54.9 ± 12.1*	34.9 ± 10.8

Pre^a^- slope (Kg/mm) for PD^b^	0.63 ± 0.12	0.59 ± 0.18

Post^a^- slope (Kg/mm) for PD^b^	0.43 ± 0.10*	0.51 ± 0.12

Pre^a^- slope (Kg/mm) for IF^c^	0.64 ± 0.05	0.52 ± 0.13

Post^a^- slope (Kg/mm) for IF^c^	0.44 ± 0.06*	0.56 ± 0.11

Pre^a^- slope (Kg/mm) for TM^d^	0.47 ± 0.15	0.46 ± 0.12

Post^a^- slope (Kg/mm) for TM^d^	0.40 ± 0.13	0.42 ± 0.12

Pre^a^-Flex-SF^e^	33.3 ± 2.8	32.6 ± 3.8

Post^a^-Flex-SF^e^	40.5 ± 5.2*	31.7 ± 3.8

Pre^a ^and Post^a^-massage *PD*^b ^posterior deltoid; *IF*^c ^infraspinatus; *TM*^d ^teres minor

*FLEX-SF*^e ^Flexilevel Scale of Shoulder Function.

*: There were significant differences between 2 groups (*P *< 0.005).

mean ± standard deviation

### Muscle tightness measurement

**Figure 2:**
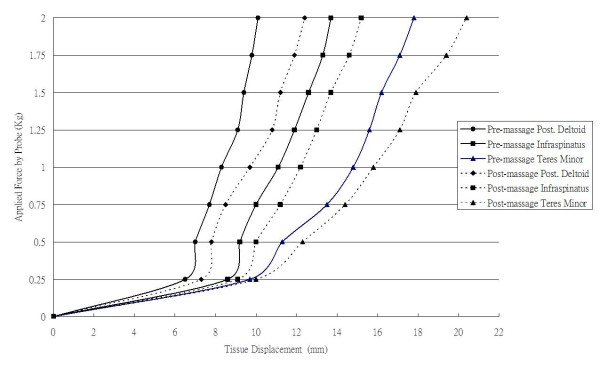
**Force-displacement curves of 3 muscle tightness**. Slopes of 3 muscles are demonstrated pre- and post-massage for one subject.

Muscle tightness, defined as the change in passive tension per unit change in length, is an indication of a muscle's passive tension to length change. The assessment of the muscle tightness can be longitudinal or transverse to the muscle [21-24]. A computerized myotonometer (Neurogenic Technologies, Inc) was used to measure the transverse tightness of the muscles. The myotonometer measures tissue tightness by quantifying the amount of tissue displacement (± 0.1 mm) as compared to the constant applied pressure as a probe is pushed downward onto the muscle and underlying tissue. The tissue displacement values were recorded at eight force probe pressures (0.25, 0.50, 0.75, 1.00, 1.25, 1.50, 1.75, 2.00 kg). The force-displacement curves were generated from these data. Thus, the slope for each force-displacement curve was calculated (Figure 2). Less penetration of the probe and a sharp slope of the force-displacement curve indicate higher resistance (more tightness). Myotonometer measurements of muscle tightness has been demonstrated to be valid and reliable [21-24]. Jenkyn et al. [25] have pointed out that transverse tightness could be correlated with muscle tension. Based on our pilot study on 8 shoulders, high intrarater within-session (20 minutes time lapsed) reliability (intraclass correlation coefficient = 0.98) of this measurement was observed. Additionally, construct validity of this measurement was observed. More posterior muscle tightness was proposed to occur in end-range position. As expected, less penetration of the probe was observed in end-range internal rotation compared to neutral internal rotation in our pilot study (*P *< 0.05).

### Functional evaluation

The self-reported Flexilevel Scale of Shoulder Function (FLEX-SF) was used to present functional disability from symptoms [26]. In this scale, respondents answer a single question that grossly classifies their level of function as low, medium, or high. They then respond to only the items that target their level of function. This scale covers the entire continuum assessment of shoulder functions and has been satisfactorily tested for appropriate psychometric properties of reliability, validity, and responsiveness to clinical change [26]. Scores were recorded from 1, with the most limited function, to 50, without any limited function in the subject. Each patient was asked to indicate functional disability at the baseline and at a 4-week follow up. The percentage change in FLEX-SF was calculated (final score - initial score)/initial score × 100). To develop a prediction method, we need to justify that the two subgroups are responsive and nonresponsive. If the change was > 20%, the patient was categorized in the responsive group. If change was < = 20%, the patient was categorized in the nonresponsive group. We chose 20% change in FLEX-SF as the responsive criterion because the patients generally felt satisfied with 20% improvement from our investigation in the clinic [27].

### Procedures

After signing the informed consent form, the subjects were examined by a physical therapist to establish the clinical conditions of their shoulders, including glenohumeral internal ROM, 3 muscle tightness measurements [posterior deltoid, infraspinatus, and teres minor muscles] and the FLEX-SF questionnaire.

In a prone position, the subject's arm was moved passively to the cessation of movement (firm end-feel) of internal rotation with the arm held in 90 degrees abduction by the tester. The recorder who was blinded to group allocation placed a hand-held goniometer (Ever Prosperous Instrument, Inc.) with two arms parallel to the forearm and trunk, respectively, and documented glenohumeral internal rotation ROM. During the test, the scapula was palpated at the lateral border and stabilized by hand. These measurements were aborted and restarted if the subject was unable to relax or if the scapula could not be stabilized effectively.

**Figure 3:**
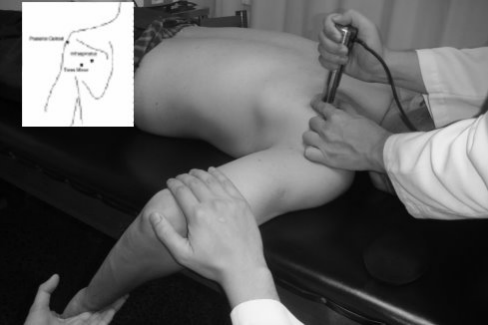
**The stiffness measurement sites for the 3 muscles using myotonometer probe placement over the 3 posterior shoulder muscles**. Posterior deltoid: two fingerbreadths caudad to posterior margin of the acromion; infraspinatus: two fingerbreadths below medial portion of spine of scapula; and teres minor: one-third of the way between acromion and inferior angle of scapula along lateral border.

Subsequently, the tightness of the 3 posterior shoulder muscles was evaluated by the assessor. Each patient was tested while maintained in internal rotation end-range prone position, and the patient was told to expose the shoulder area undergoing the testing. The patient was asked to relax the shoulder. A surface electromyography was used to monitor the muscle tone and to confirm muscular activity at rest (less than mean activity plus 2 standard deviation at rest for 1 minute with shoulder neutral rotation in prone position) during muscle tightness measurement. The head of the myotonometer probe was placed over the 3 posterior shoulder muscles in Latin square order (posterior deltoid: two fingerbreadths caudad to the posterior margin of the acromion; infraspinatus: two fingerbreadths below the medial portion of the spine of the scapula; teres minor: one-third of the way between the acromion and the inferior angle of the scapula along the lateral border). The placements of probe head were between 2 electrodes of EMG of each muscle to confirm resting muscular activity during muscle tightness measurement. According to the software manual, each muscle was tested in three trials (each trial had 4 measurements) (Figure 3). Each muscle was tested in three trials (each trial consisted of 4 measurements). Myotonometer data recordings of all eight force increments were acquired in approximately 1 second. The intrarater/interrater reliabilities are high (ICC = 0.99) on muscle tightness measurements [28]. Therefore, the mean of 3 trials for each muscle was calculated for data analysis.

For the massage group, 2 physical therapists with at least 8 years of clinical experience in manual therapy provided the massage on the posterior deltoid, infraspinatus, and teres minor of the involved shoulder for 18 minutes (about 6 minutes for each muscle with Latin square order) two times a week for 4 weeks. The techniques of massage including petrissage for 3 minutes and rolling for 3 minutes of soft tissues were applied to the patients with prone position and arm by side [29]. For the control group, same therapists applied light hand touch on the muscles (placebo control) 10 minutes two times a week for 4 weeks. After 4 weeks, the glenohumeral internal rotation ROM and 3 muscle tightness measurements at the pre-massage internal rotation position (posterior deltoid, infraspinatus, and teres minor muscles) were evaluated by the same blinded assessor for each patient.

### Data analysis

Data were analyzed using SPSS 15 software (SPSS Inc., Chicago, IL). Baseline variables were compared between groups using independent t tests. To test whether a difference of treatment efficacy existed between 2 groups, 2-factor ANOVA mixed models with factors of group (control group, massage group) and time (the initial data and follow up data at 4 weeks) were performed on each of the outcomes. Bonferroni follow-up analyses were used to adjust for multiple pair-wise comparisons where appropriate. Intention-to-treat analysis was performed by including the drop-out data carrying the last data point forward into analysis. Additionally, Pearson correlations between tightness slope for each muscle and BMI were calculated to evaluate the potential skin/subcutaneous tissue thickness effect on the muscle tightness measurement.

We evaluated the potential predictors for the massage treatment. Responders versus non-responders within the massage group were compared with the chi-square or *t *test for all potential predictor variables (sex, age, BMI, duration of symptoms, glenohumeral internal rotation, muscle tightness in each muscle, and FLEX-SF score), as appropriate. Predictor variables that had a difference with a p-value ≤ .10 were entered into a logistic regression model. The variables with the least predictive value were then removed, one by one, in a backwards stepwise fashion until all predictors in the model had *p*-values ≤ 0.05.

## Results

Fifty-two patients completed the study (29 for the massage and 23 for the control). One subject in the massage group failed to attend the treatment. In the control group, seven subjects were lost to follow up. Baseline variables were not significantly different between groups (*p *> 0.05) (Table 1). The overall mean internal rotation ROM increased significantly in the massage group compared to the control (54.9° v.s. 34.9°; *P *≤ 0.001). The overall mean FLEX-SF increased significantly in the massage group compared to the control (40.5 v.s. 31.7; *P *≤ 0.001). The overall 3 mean muscle tightness decreased significantly in the massage group compared to the control (0.42 v.s. 0.51; *P *≤ 0.05). Similar results were found for intention-to-treat analysis (inclusion of drop-out data). There were no significant correlations between tightness slope for each muscle and BMI (R range between -0.19 to 0.23, *p *> 0.05). Thus, the potential skin/subcutaneous tissue thickness confounding effect on the muscle tightness measurement was not likely to occur in our samples.

For the massage group, the mean glenohumeral internal rotation ROM was 31.9° before massage, and significantly improved to 54.9° after massage (*P *< 0.001). Among these patients, 21 were classified as responsive and 8 as nonresponsive. The responsive group had significantly less duration of symptoms, more mean posterior deltoid and infraspinatus slopes (more tightness before massage), and larger FLEX-SF scores (less limited function) than the nonresponsive group (Table 2).

**Table 2:** Subject demographics

variable	Responsive (n = 21)	Nonresponsive (n = 8)
Gender (males:females)	6:15	2:6

Age (years)	55.3 ± 5.7	52.5 ± 7.8

Height (cm)	162.8 ± 6.8	165.4 ± 7.2

Weight (Kg)	65.4 ± 7.9	63.3 ± 5.8

Duration of symptoms	11.7 ± 3.4*	17.9 ± 4.8

Pre-Glenohumeral Internal rotation (°)	32.1 ± 9.5	26.1 ± 6.6

Post-Glenohumeral Internal rotation (°)	68.8 ± 12.1*	32.2 ± 10.8

Pre-slope (Kg/mm) for PD^a^	0.62 ± 0.11*	0.56 ± 0.09

Post- slope (Kg/mm) for PD^a^	0.42 ± 0.09*	0.52 ± 0.13

Pre- slope (Kg/mm) for IF^b^	0.63 ± 0.02*	0.48 ± 0.09

Post- slope (Kg/mm) for IF^b^	0.48 ± 0.07*	0.52 ± 0.10

Pre- slope (Kg/mm) for TM^c^	0.49 ± 0.15	0.48 ± 0.11

Post- slope (Kg/mm) for TM^c^	0.41 ± 0.13	0.39 ± 0.11

Pre-Flex-SF^d^	32.9 ± 2.5	32.5 ± 6.1

Post-Flex-SF^d^	43.3 ± 4.8*	38.2 ± 2.8

*PD*^a ^posterior deltoid; *IF*^b ^infraspinatus; *TM*^c ^teres minor

*FLEX-SF*^d ^Flexilever Scale of Shoulder Function.

*: There were significant differences between 2 groups (*P *< 0.005)

The logistic regression analysis for effectiveness variables showed that duration of symptoms, FLEX-SF score, and posterior deltoid slope correlated with the effectiveness of massage (*P *< 0.05) (Table 3). The patients with less duration of symptom, higher FLEX-SF scores (less limited), and higher slope of posterior deltoid (more tightness) increased the possibility of effective massage in the treatment of patients with posterior shoulder tightness.

**Table 3:** Logistic regression with stepwise method analysis at baseline data

**Predictor**	B	SE	Adjusted odds ratio	95% CI for odds ratio	P value
Duration	-0.31	0.13	0.72	0.55-0.98	0.012

FLEX-SF^a ^score	0.90	0.26	2.44	1.39-4.39	0.002

PD^b ^slope	5.71	5.40	300	3.36-300	0.018

*FLEX-SF*^a ^Flexilevel Scale of Shoulder Function

*PD*^b ^Posterior Deltoid

With a 1 month difference in duration, such as from 11 to 12, the odds of being responsive or nonresponsive are essentially even [adjusted odds ratio = 0.72]. With a one score difference in FLEX-SF, such as from 27 to 28, the odds of being responsive were almost 2.4 times greater for someone who is 28 as compared to someone who is 27 [adjusted odds ratio = 2.44]. With a 0.1 difference in slope of posterior deltoid, such as from 0.4 to 0.5, the odds of being responsive were almost 1.8 times greater for someone who is 0.5 as compared to someone who is 0.4 [adjusted odds ratio = 300]

## Discussion

In this study, 4-week massage was effective in internal rotation ROM, FLEX-SF, and muscle tightness compared with the control group. We also investigated the predictors of the effectiveness of massage to treat subjects with posterior shoulder tightness. Because internal rotation ROM limitation and functional disability is a common limitation for subjects with posterior shoulder tightness [15,30]. We assessed the effectiveness of massage by change in internal rotation ROM and the FLEX-SF scores. The minimum detectable significant change in internal rotation ROM has been reported as 4° over 1 week [31]. This value, however, may vary with the limited status of patients. In our study, the improvement of overall mean internal rotation ROM by massage was 20.4° at 4 weeks, where the initial internal rotation ROM (31°). Our 4-week massage provide an approximate 66% improvement of limited internal rotation ROM. indicating that 16.5% (5°) improvement for one week massage. This result was comparable with the previous reports [3,17,32].

Among the complex variables that were proposed to contribute to subjects with posterior shoulder tightness [33], we focused muscular theories and selected those that could be easily determined and quantified clinically as the objects of this investigation. In agreement with the statements of previous studies, that the posterior muscles play a role in posterior shoulder tightness [11,12], our results specifically indicated that the 3 muscles made contributions to glenohumeral internal rotation ROM. Additionally, we found that duration of symptoms, FLEX-SF scores, and posterior deltoid tightness were predictive of effective massage.

Subjects with different severity of restricted ROM are likely to respond differently to massage. In our sample with restricted internal rotation ROM of 31°, improvement of internal rotation ROM was more obvious after massage in the posterior deltoid than in the other two muscles. On the other hand, results from a previous study [17] indicated improvement from massage on infraspinatus and teres minor muscles in a case with restricted internal rotation ROM 68°. Thus, tightness reduction in different muscles may contribute to improvement of internal rotation ROM. Additionally, this phenomenon could be associated with muscle anatomical structures. Liu et al. [32] indicated that role of deltoid varies according to different parts of the muscles. Posterior deltoid is believed to play an adduction component during abduction. Thus, the posterior deltoid may be the first component to restrain the internal rotation measurement in abduction position, as in our measurement. Further research needs to examine this phenomenon.

The mechanism of internal rotation ROM limitation may be complex. Studies in the literature have proposed a dual mechanism, including a capsular tightness that results in rotation and abduction ROM limitation of shoulder joint [4], and a muscular tightness that specific muscular tightness is related to glenohumeral internal ROM limitations [11,12,32]. This study did not intend to investigate the mechanisms of massage on internal rotation ROM, but attempted to find the characteristics of patients responsive to massage. We found that subjects with less duration of symptoms, higher functional status (high FLEX-SF scores), and more tightness in posterior deltoid have higher possibility of being responsive to massage treatment.

Limitations of the study should be noted. Myotonometer measurements can be compromised by the possibility of other soft tissue in addition to the target muscle. We believe that the technique (6 minutes of massage for each muscle in our study) treated mainly the target muscle, with only minor stressing of the other muscles or joint capsule. Because the 3 muscles are close to each other, the soft tissues compositions above the 3 muscles should be similar. The different tightness values among the 3 muscles in our study indicated that measurement with a Myotonometer focused on the tightness characteristics of each target muscle. Additionally, low correlation between tightness slope for each muscle and BMI confirmed our measurements. The skin/subcutaneous tissue thickness was not likely to affect our results.

## Conclusions

Muscle tightness reduction in the posterior shoulder contributes to improvement of glenohumeral internal rotation ROM after 4-week massage. These effects varied among the posterior deltoid, infraspinatus, and teres minor muscles. Additionally, less duration of symptoms and higher functional status of the subject can predict the effective massage treatment.

## Competing interests

The authors declare that they have no competing interests.

## Authors' contributions

J-JL carried out the research design studies and coordination, participated in the subject assignment, performed the statistical analysis, and drafted the manuscript. J-LY carried out the treatments and helped to draft the manuscript. C-LH and S-YC participated in the research design and helped to draft the manuscript. All authors read and approved the final manuscript.

## Note

This study is supported by NSC-2314-B-002-006-MY3 and NTUH-99S1339.

## Pre-publication history

The pre-publication history for this paper can be accessed here:

http://www.biomedcentral.com/1471-2474/13/46/prepub
